# Factors Associated with Primary School Teachers’ Attitudes Towards the Inclusion of Students with Disabilities

**DOI:** 10.1371/journal.pone.0137002

**Published:** 2015-08-28

**Authors:** Sharmila Vaz, Nathan Wilson, Marita Falkmer, Angela Sim, Melissa Scott, Reinie Cordier, Torbjörn Falkmer

**Affiliations:** 1 School of Occupational Therapy and Social Work, Curtin University, Perth, Western Australia, Australia; 2 School of Nursing and Midwifery, University of Western Sydney, Sydney, New South Wales, Australia; 3 School of Education and Communication, CHILD programme, Institution of Disability Research Jönköping University, Jönköping, Sweden; 4 Rehabilitation Medicine, Department of Medicine and Health Sciences (IMH), Faculty of Health Sciences, Linköping University & Pain and Rehabilitation Centre, UHL, County Council, Linköping, Sweden; TNO, NETHERLANDS

## Abstract

**Objective:**

Teachers' attitudes toward inclusion are often based on the practical implementation of inclusive education rather than a specific ideology and understanding of inclusiveness. This study aimed to identify the factors associated with primary school teachers' attitudes towards inclusion of students with all disabilities in regular schools.

**Method:**

Seventy four primary school teachers participated in a cross-sectional survey conducted in Western Australia. Teachers' attitudes and efficacy toward integration of students with disabilities were measured using the Opinions Relative to Integration of Students with Disabilities scale and Bandura's Teacher Efficacy scale respectively.

**Results:**

Four teacher attributes—age, gender, teaching self-efficacy and training collectively explained 42% of the variability in teachers' attitude toward including students with disabilities.

**Conclusion:**

The current study further contributes to the accumulation of knowledge that can unpack the complex pattern of factors that should be considered to promote positive attitudes towards inclusive schools.

## Teachers’ Attitudes Towards Inclusion

The Salamanca Statement highlights the need to provide education for all children in an inclusive school [1]. As a result, the implementation of inclusive schools has been a goal in many countries [2]. Inclusion is based on the concept of social justice; wherein all students are entitled to equal access to all educational opportunities, irrespective of disability or any form of disadvantage [3]. In Australia, the Commonwealth and State educational governments advocate for the inclusion of children with disabilities within regular classrooms [4–6]. Nevertheless, advocacy alone does not ensure that the policy is favourably accepted by those on the frontline of implementation, namely, classroom teachers. Studies have revealed that teacher attitudes and expectations are significant barriers to the successful implementation of inclusive classrooms [7–9] and equitable participation of all students [10].

Attitudes are conceptualised as relatively stable constructs comprising cognitive, affective and behavioural components [11]. Teachers’ attitudes towards inclusion are often based on practical concerns about how inclusive education can be implemented, rather than be grounded in any particular ideology [12, 13]. Common practical concerns raised by teachers include: accommodating the individualised time demands of students with disability without disadvantaging other students in the classroom; being apprehensive of the quality and quantity of work output of children with disabilities; lacking adequate support services; and limited training and competence in supporting inclusive educational practice [14].

The severity of the disability that teachers are required to accommodate within their classroom is inversely associated with their attitude towards inclusion. That is, the more severe the child's disability; the less positive their attitude is towards inclusion [15–18]. The type of disability also appears to influence teachers’ attitudes. For example, teachers were found to generally be more supportive of including children with physical and sensory disabilities than those with intellectual, learning and behavioural disabilities [7, 8, 16, 18, 19].

Teacher education is viewed to be pivotal in developing the affirmative attitudes and skills required for successful inclusion, with formal educational training being identified as one of the main factors that promote an inclusive attitude [14, 20–22]. Similar findings have been found with trainee teachers [21, 23], where the inclusion of a compulsory module on diversity in a post-graduate degree promoted having an inclusive attitude. Pedagogies that combine formal training and planned hands-on experience with people with disabilities have been shown to improve preparedness and positive attitudes towards inclusion [7, 24, 25]. Moreover, irrespective of degree type, trainee teachers had a better understanding of the potential of children with disabilities after completing a unit of study with a strong focus on inclusive education [24]. However, some authors argue that improving knowledge of and confidence in inclusive education alone is insufficient in improving a positive attitude towards inclusion and reducing related anxiety. They highlight the finding that there is a gradual decline of positive attitudes towards inclusion in trainee teachers as they advance in their training years [23, 26]. Perhaps an increased awareness of the challenges one is likely to face by including all students with disabilities might dampen teachers’ openness towards being inclusive [15, 27].

The influence of age, gender and role on having an inclusive attitude is largely mixed. Some studies reported no significant effect of teachers’ age on having an inclusive attitudes [7, 18], while others suggest training in inclusive practices significantly improves the attitudes of younger trainee teachers, but not older ones [21]. Female teacher trainees are reported to be more tolerant in implementing inclusive education [18, 28]; while other studies reported no effect of gender [29–31]. Following training, teachers with less experience have been shown to have a more positive attitude towards inclusion when compared with their more experienced counterparts [7, 8, 32]. Conversely, some studies found that teachers who have been exposed to people with disabilities (i.e., friend or family member) were found to be more open to inclusion [21, 33, 34], whereas other studies do not report any influence of prior exposure to disability [29]. A recent cross-cultural study on trainee teachers’ attitudes toward multiple aspects of diversity found that overall attitudes toward people who differed from them were ‘predominantly acceptance’ regarding disability, gender and special talents; with over 80% of the participating trainee teachers upholding a positive attitude [35].

In recent years, there has been growing interest in studying the pragmatic side of implementing inclusive education by measuring teachers’ sense of self-efficacy of implementing inclusive education [36]. Self-efficacy in teaching is the belief that one’s teaching can influence how well all students learn, including those who are unmotivated or demanding [36, 37]. The importance of self-efficacy emerges from its cyclic nature, whereby proficiency in performance creates a new mastery experience which, in turn, influences self-efficacy beliefs [38]. Empirical findings validate the associations between high self-efficacy in teachers and openness to implement varied instructional strategies for students of all ability levels, including those with learning difficulties [39] and more positive attitudes toward inclusive education [40–42]. Conversely, teachers with low self-efficacy in their teaching are more likely to see difficulties in learning to be attributable to the child (i.e., internal to the child) and less willing to adapt their teaching methods to suit the needs of students with learning difficulties [43–46]. Teachers with a higher efficacy attribute students’ difficulties more to external factors than those with a lower efficacy, suggesting that teachers who feel more competent are more comfortable in accepting some responsibility for students’ difficulties [43]. Emerging evidence suggests that teachers’ self-efficacy beliefs are a better predictor of the attributes they uphold regarding inclusive education than their role (i.e., whether a teacher works in a special, mainstream, or learning support setting) [43].

Thus, while the impact of teacher attitudes on the implementation of inclusion policies is widely recognised, the factors shaping these attitudes are poorly understood. The current study aimed to identify the factors associated with primary school teachers’ attitudes towards inclusion of students with all disabilities in mainstream schools in Western Australia (WA).

## Method

### Design

The current study reports data obtained from a cross-sectional survey. The study is part of a larger longitudinal study investigating the factors associated with academic, social, emotional and mental health outcomes of students with and without disabilities as they transition from primary to secondary school [47, 48]. Recruitment for the current study was extended to 250 mainstream primary schools listed on the Department of Education and Training, WA website. Schools listed in the Canning, Fremantle-Peel, Swan, and West Coast educational districts of Perth and major centres of Albany, Bunbury, Mid-West, Midlands, and Esperance educational districts of WA were approached. Several recruitment strategies were used to maximise reach and representativeness.

Classroom teachers in charge of students in the final year of primary school in WA (class 6 or 7) in the academic years commencing January 2006 or 2007, and due to transition to either middle or secondary school in January 2007 or 2008 were eligible to participate in the study. These included teachers who catered for students with a disability or chronic condition who attended a regular class for the majority of their weekly schooling hours (over 80% of the school hours per week), with classroom support provided as required. The predominant disabilities included auditory and visual disability, learning disability, Attention Deficit Hyperactivity Disorder, Autism Spectrum Disorders, and Cerebral palsy. The chronic conditions included asthma, diabetes and thyroid dysfunction.

The response rate was 30%. Cross-sectional data were collected from 74 primary school teachers across 74 schools in inner city and regional areas of WA. Information was collected via survey questionnaires, primarily in paper and pencil format. Details on the study’s design, recruitment, and data collection have been published elsewhere [47, 48]. Informed written consent was obtained from school principals and teachers to participate in this study. Participation in the study was voluntary. Participants were made aware that they were not obliged to participate in the study and were free to withdraw from the study at any time without justification or prejudice. The study received ethical approval from the Curtin University Human Research Ethics Committee (HR 194/2005) in Perth, WA.

### Data collection instruments

#### Opinion relative to integration of students with disabilities

The Opinions Relative to Integration of Students with Disabilities scale (ORI) was the chosen outcome measure [49]. This rating scale measures teachers' attitudes toward the integration of students with disabilities in regular settings by presenting statements such as: *“Integration of special needs students will require significant changes in regular classroom procedures*,*”* or *“The integration of special needs students can be beneficial for regular students*.*”* The ORI contains 25 positively and negatively worded statement options rated on a 7-point Likert scale, with responses to the items ranging from -3 (I disagree very much) through +3 (I agree very much) [49]. The ORI is reported to have high internal consistency (Cronbach’s *α* = .88) and split-half reliability (Spearman-Brown prophecy = .82) [49]. Moderate concurrent validity has between reported between the ORI score and the Scale of Attitudes towards Disabled Persons (*r* = .66). In the current study, the ORI scale was used as a uni-dimensional construct; with higher scores indicating poorer attitude to inclusion. The internal consistency of the ORI score in the current sample was high (Cronbach’s *α* = .92).

#### School and teacher characteristics

Teachers reported details on the demographic characteristics, education, training and general characteristics of the school. Information on the school sector, post codes of students enrolled in each school and organisational structure at each school was obtained from Department of Education and Training (WA) records. The school’s post code was used to calculate its socio-economic index (SEIFA Index), using the Commonwealth Department of Education, Employment, and Workplace Relations measure of relative socio-economic advantage and disadvantage [50]. The SEIFA decile was used as the measure of mean school-SES, with a lower decile ranking being indicative of greater disadvantaged relative to high decile rankings which are indicative of greater affluence.

#### Bandura’s Teacher Efficacy scale

The 30-item Bandura’s Teachers Efficacy scale was used to assess teachers’ efficacy beliefs [51, 52]. The scale measures perceived efficacy to influence: decision making; use of school resources; instructional and disciplinary practices in school; enlist parental involvement; enlist community involvement; and creation of a positive school climate. Measurements are anchored on a nine-point scale, with notations ranging from ‘nothing, very little, some influence, quite a bit, and a great deal’. Items are scored such that a higher score indicates greater efficacy. In the current study, mean teacher efficacy was computed. The average score for the 30-item score had strong internal consistency; with Cronbach’s α values of .92 (Hoy, 2000) and .94 in the current study.

### Data Analysis

Data were analysed using the Statistical Package for the Social Sciences (SPSS Version 20) and Statistical Analysis System (SAS Version 9.2) software. Only 1.8–2.5% of data were missing at scale levels. The estimation maximization (EM) algorithm and Little’s chi-square statistic identified data to be missing completely at random, with the probability level set at .05 [53, 54]. Standard guidelines recommended by instrument developers were followed to replace missing values. Where guidelines were not present, missing values were replaced by mean scores [55]. Independent samples *t—*tests confirmed that the characteristics of those whose data were missing for various questions were similar to those who responded.

Descriptive statistics were used to summarise the profiles of study participants. Multiple linear regression models were run to describe the relations between school, classroom and teacher factors (Independent variables, IV) and the teachers’ attitude to inclusion scores (Dependent variables, DV). Dummy variables were created to represent categorical IVs being incorporated into the regression models. In the case of continuous IVs (e.g., teachers’ efficacy scores) the score was divided into quartiles [56]. General Linear Model Analysis of Variance (ANOVA) were conducted to test whether the DV appeared to vary in a linear fashion across the four quartile categories of the IVs based on the marginal mean estimates.

## Results

### Descriptive characteristics of the participating teachers and schools

Teachers involved in this study represented 74 WA mainstream primary schools. As shown in Table 1, the majority of the schools came from higher decile regions of WA, and over mid-range in student size. Less than 20% of the teachers taught classrooms with less than 25 students.

**Table 1:** Characteristics of the schools and classrooms.

School characteristics	IV categories	n (%)
**School sector**	Government	37 (50%)
	Catholic	25 (33.8%)
	Independent/Private	12 (16.2%)
**Mean school SES**	1–6 (lower decile range)	20 (27%)
	7–8 (mid decile range)	12 (16.2%)
	9–10 (upper decile range)	42 (56.8%)
**School size based on total no of students**	Small: < 375 students	20 (27%)
	Mid-range: 375–975 students	45 (60.8%)
	Large: > 975 students	9 (12.2%)
**Class-room size**	Small: < 25 students	14 (18.9%)
	Mid-range: 25–30 students	32 (43.2%)
	Large: > 31 students	28 (37.8%)
**Co-education classes**	Yes	66 (89.2%)
	No	8 (10.8%)
**No. of students with disability in school**	None	38 (51.3%)
	One or more	36 (48.7%)

Note: IV is used as an abbreviation of independent variable.

As shown in Table 2, there was a fairly even distribution of the teachers by gender. Almost two-thirds of the teachers were in the 35–55 year age bracket, and more than 85% worked full-time. In terms of qualifications, less than a quarter of teachers held degrees in inclusive education. Less than half of the teachers reported attending professional development training on inclusive teaching practices during the school year of the study. The participating teachers varied in teaching experience, with the majority having between 11–30 years, and a minority less than 10 years of teaching experience. Furthermore, teachers varied in their experience in teaching students with a disability, with less than a quarter reporting no experience in teaching students with a disability, and a third reporting three or more years of experience. A quarter of the involved teachers reported low levels of self-efficacy in general teaching.

**Table 2:** Characteristics of teachers involved in the study.

Teacher demographic factors	IV categories	n (%)
**Gender**	Female	42 (56.8%)
	Male	32 (43.2%)
**Teacher age**	< 35 years	11 (18%)
	35–55 years	39 (63.4%)
	55years and over	11 (18%)
**Employment status**	Full-time	65 (87.8%)
	Part-time	9 (12.2%)
**Degree or post-graduate degree in inclusive teaching**	No	56 (75.7%)
	Yes	18 (24.3%)
**Attended professional development courses related to teaching students with disability**	No	36 (48.6%)
	Yes	38 (51.4%)
**Teaching experience**	< 10 years	19 (26%)
	11–30 years	40 (54.8%)
	> 31 years	14 (19.2%)
**Years of experience in teaching students with disability**	No experience	12 (20%)
	1–2 years	27 (45%)
	3 years and more	21(35%)
**Self-efficacy in teaching**	Low-quartile	65 (25%)
	Middle-half	138 (53.1%)
	High-quartile	57 (21.9%)

### Factors predicting teachers’ attitude towards inclusion of students with disabilities

A preliminary screening was conducted through examination of residuals. At each step of the multiple regression analyses, the scatterplot of residuals against predicted values was examined. No multivariate outliers were found in any of the steps [54]. No obvious pattern to the errors was detected through examination of the residual scatterplots. Table 3 shows the unstandardised regression coefficients (B) and standard errors (SE), and the standardised regression coefficients (Beta), after entry of all variables. R was significantly different from zero at the end of each step. No significant interactions were found, so they were removed from the models.

**Table 3:** Factors predicting teachers’ attitude to inclusion.

Model	Unstandardized Coefficients		Standardised Coefficients	*t*	*p*	95% Confidence Interval for B	
	B	Std. Error	Beta			Lower Bound	Upper Bound
**(Constant)**	127.94	4.08		31.29	.000	119.71	136.17
**Small class vs. mid-sized class**	10.35	5.30	.25	1.95	.057	-.32	21.03
**Large class vs. mid-sized class**	-8.53	4.51	-.25	-1.89	.065	-17.63	.55
**Male teacher vs. female teacher**	-8.66	4.06	-.26	-2.13	.038	-16.85	-.48
**Age >55 years vs. 35–55 years**	-24.65	7.31	-.55	-3.36	.002	-39.39	-9.92
**Training in teaching students with disability**	12.28	5.56	.28	2.20	.032	1.09	23.48
**>31 years’ experience in teaching**	18.27	7.10	.44	2.57	.013	3.96	32.57
**Low levels of self-efficacy in teaching Vs. Mid-level self-efficacy in teaching**	-13.74	4.37	-.38	-3.14	.003	-22.54	-4.94

Four teacher attributes—age, gender, teaching self-efficacy, training—collectively explained 42% of the variability in teachers’ attitude towards including students with disabilities (F _(7, 46)_ = 4.37, *p* < .001).

1. Male teachers had a more negative attitude towards inclusion (Beta = -.26, *p =* .04).
2. Teachers who were aged 55 years and over upheld more negative attitudes towards inclusion when compared to the 35–55 year old subgroup (Beta = -.55, *p =* .002).
3. Teachers with low-levels of self-efficacy in their teaching skills were more likely to also uphold negative attitude towards including students with disabilities (Beta = -.38, *p =* .003).
4. Teachers who reported having training in teaching students with disability upheld positive attitudes towards inclusion (Beta = .29, *p* = .032).

Of note is that school factors (including, type of school, SEIFA index, sector, and size); classroom attributes; teacher attributes (including, type and level of education degree, receipt of education in inclusive teaching practices, and years of experience in teaching students with disabilities); and student characteristics such as gender and whether the child received support in academics or non-academic areas of schooling, did not significantly influence the attitudes of the teachers towards inclusiveness.

## Discussion

There appears to be broad consensus that teachers’ attitudes toward inclusion is critical in implementing the ambitious goal of inclusive schools and for these strategies to be successful [7]. Attempts to identify factors associated with teachers’ attitudes toward inclusion has been mixed so far, albeit some notable trends suggesting that severity of disability, the availability of support and perceived competence are all important factors. Hence, results from the current study further contribute to the accumulation of knowledge that can unpack the complex pattern of factors that should be considered to promote positive attitudes towards inclusive schools [57]. Importantly the current study provides greater insights into the significance of gender, age, teaching self-efficacy, and targeted training on attitudes towards teaching students with disabilities.

Previous studies have presented mixed results regarding the impact of gender on attitudes towards inclusion. Results from studies conducted in the 1990s summarised in a literature review show that in four out of seven studies, female teachers held more positive attitudes toward inclusion than male teachers [7]. In a later review, two out of three studies reported the same results, i.e., that female teachers were more positive towards inclusion compared with their male colleagues [8, 58]. The results from the current study add to the evidence that gender appears to be a predictor of teacher attitudes towards inclusion and that male teachers tend to have a more negative attitude than female teachers. While the reason for this gendered difference remains open to conjecture, researchers have attributed this reported disparity to a greater tolerance and more conative attitude for inclusion in females [7, 28]. That is, there may actually be no difference between male and female teachers in the actual practice of inclusive education, rather than contemplating the idea of inclusion.

The results of the current study aligned with previous research indicating that older teachers tend to have more negative attitudes towards inclusion [7, 8]. This may not be a surprise, as older teachers are to have had limited or no training in inclusive teaching. Hence, these teachers may have to adapt not only to a new group of students that requires additional support and alternative teaching strategies, but also to an inclusive school as a new concept that might differ from the school they envisioned themselves working in.

It has been reported that a lack of confidence in regard teaching students with special needs were associated with negative attitudes to inclusion [59]. Hence, teachers attitudes are probably related to how much the struggle with identifying solutions to problems—such as the availability of human, physical and environmental supports, being able to accommodate students with severe disabilities and lacking the required skills to deal with students with a disability [7].

Knowledge appear to be a key factor that influences teachers’ ability to change teaching practices [60]. Teachers’ knowledge can be divided into content knowledge (i.e., knowledge about the subject), pedagogical knowledge (i.e., including teaching and classroom management strategies), and pedagogical content knowledge (i.e., how to teach a particular content to a specific student in a defined context) [61]. Training in teaching students with disabilities was associated with positive attitudes towards inclusion. However, upholding a degree in inclusive teaching did not contribute to the model. It could be of interest to further examine if training specifically designed to prepare teachers to teach students with disabilities may be better at incorporating all aspects of knowledge compared with the formal training in inclusive teaching. The fact that low self-efficacy in teaching skills was associated with negative attitudes in the current study, as well as in previous studies [36, 40, 62], further emphasises the importance of focusing on teachers’ knowledge. In particular, a focus needs to be placed on enhancing pedagogical content knowledge related to students with disability when aiming to positively influence teachers’ attitudes towards inclusion. Additionally, knowledge about specific disabilities and/or conditions has been reported to positively impact attitudes [8]. This indicates that overall strategies for inclusive teaching may not be sufficient and that pedagogical content knowledge may be enhanced by insight in to specific diagnoses and how they can affect students and their learning.

## Limitations

The sample included teachers in regular schools from the Perth metropolitan area and major city centres across WA, and did not involve teachers from regional or remote populations, or other inner city areas in Australia. Despite extensive recruitment efforts, 70% of the 250 schools declined to participate in this study, which may have introduced a possible bias. However, it is not possible to decide whether the participating schools were negatively or positively biased towards inclusion of students with disabilities. The findings of the current study are based on a cross-sectional data; therefore, no causality should be assumed. From a methodological point of view, there might be other models with other predictors as plausible as the ones presented. Although the measures used in the current study were psychometrically sound; they do not provide information that can explain why teachers uphold the attitudes they do. This would require in-depth analyses of teachers’ perceptions. We did not include factors, beyond the classroom, such as: teachers’ general attitudes towards people with disabilities in the wider society [43, 63]; and the cognitive and behavioural processes teachers’ bring into play in coping with perceived stress [64, 65]. Other studies have suggested that barriers and facilitators towards acceptance of diversity and inclusion are embedded within the social and cultural contexts in which an individual is situated [66]. Future cross-cultural studies are needed to understand these facilitators and barriers in order to improve the inclusive practices of all teachers.

## Future Directions

Given the limitations of the current study, a clear future direction is to collect inductive and longitudinal data on the development and change of teachers’ attitude towards inclusion. Longitudinal data would enable analysis using cross-lagged prediction models and measuring stability of these constructs over time. Future research needs to unpack in greater detail some of the deeper issues relating to gender of the teacher, as it is certainly too simplistic to simply state that male teachers have more negative attitudes towards inclusion. Areas for inquiry include factors raised by Cushman [67] such as: salary; working in a female-dominated sector; and concerns over physical contact with children with disabilities. For example, other research on gender differences in interacting with and supporting people with disabilities have uncovered a range of specific issues, such as personal care, support for sexual health and working in a female dominated environment [68, 69], that warrant further attention. In summary, we return to an earlier comment that teachers may not hold overtly negative attitudes; instead differences in self-efficacy may simply mean that some teachers struggle to identify solutions to problems beyond their circle of control [7, 59].
